# Laser Scanning Confocal Thermoreflectance Microscope for the Backside Thermal Imaging of Microelectronic Devices

**DOI:** 10.3390/s17122774

**Published:** 2017-11-30

**Authors:** Dong Uk Kim, Chan Bae Jeong, Jung Dae Kim, Kye-Sung Lee, Hwan Hur, Ki-Hwan Nam, Geon Hee Kim, Ki Soo Chang

**Affiliations:** Division of Scientific Instrumentation, Korea Basic Science Institute, 169-148 Gwahak-ro, Yuseong-gu, Daejeon 34133, Korea; dukim2013@kbsi.re.kr (D.U.K.); jcb5102@kbsi.re.kr (C.B.J.); kjd1105@kbsi.re.kr (J.D.K.); kslee24@kbsi.re.kr (K.-S.L.); hurhwan@kbsi.re.kr (H.H.); namkihwan@kbsi.re.kr (K.-H.N.); kgh@kbsi.re.kr (G.H.K.)

**Keywords:** thermal imaging, reflection, confocal microscopy

## Abstract

In this paper, we report on a confocal thermoreflectance imaging system that can examine the thermal characteristics of microelectronic devices by penetrating the backside of a device through the substrate. In this system, the local reflectivity variations due to heat generation in the device are measured point by point by a laser scanning confocal microscope capable of eliminating out-of-focus reflections and the thermoreflectance is extracted via Fourier-domain signal processing. In comparison to the conventional widefield thermoreflectance microscope, the proposed laser scanning confocal thermoreflectance microscope improves the thermoreflectance sensitivity by ~23 times and the spatial resolution by ~25% in backside thermoreflectance measurements.

## 1. Introduction

Thermal management is an important issue for microelectronic device design because local excess heating degrades the device performance, reliability, and lifetime. Thermoreflectance microscopy (TRM) is a well-established thermal imaging technique that provides non-contact, non-invasive measurements with high spatial and thermal resolutions [1,2,3,4,5]. In the conventional approach, TRM employs widefield illumination and detection using the imaging optics of a widefield microscope, visible (VIS) light-emitting diodes, and a Si charge-coupled device (CCD) camera. This technique can be used to image the surface thermal distributions of active devices because it can detect the relative variations in the reflectivity of a sample in response to variations in the temperature:

(1) $$\frac{\Delta R}{R}=\frac{1}{R}\frac{\partial R}{\partial T}\Delta T=\kappa \;\Delta T$$

where *R* and *T* are the reflectivity and temperature on the surface of the sample, respectively and *κ* is the thermoreflectance coefficient, which is on the order of about 10^−5^–10^−3^ K^−1^. This coefficient strongly depends on not only the material and composition of the sample but also the instrument configuration, such as whether a widefield or point illumination method is employed, the illumination wavelength and the numerical aperture of the objective lens [1].

In modern high-density microelectronic devices, a flip-chip configuration consisting of multilevel metal layers is now commonly used. Consequently, the hidden active semiconductor region can only be accessed on the backside through a Si substrate. At the same time, the TRM technique has advanced and now can be used to investigate the thermal characteristics of buried semiconductors devices inside integrated circuits [6,7]; however, there are two major challenges for the backside thermal imaging of flip-chip bonded devices, namely, the measurement resolution and sensitivity. The measurement resolution is limited by the spatial resolution due to the relatively long wavelength (>1.1 μm) that is required to penetrate the Si substrate with a low absorption, while the sensitivity is limited by extraneous reflections from the interface between the air and Si substrate. These reflections serve to reduce the variation of the relative reflectivity (Δ*R*/*R*) because they are coincident with the reflections from the region of interest (ROI) inside the substrate. Consequently, the extraneous reflections decrease both the contrast and signal-to-noise ratio (SNR) of the thermoreflectance (Δ*R*/*R*) measurements. In addition, coherent illumination methods, such as near-infrared (NIR) lasers, are subject to interference due to multiple reflections between the front and bottom of the Si substrate, which can result in the distortion of Δ*R*/*R* [7]. In a previous work, a TRM system for backside thermal imaging was designed that was similar to VIS TRM, except for the use of incoherent NIR illumination and a solid immersion lens (SIL) [8,9]. When tested, the system was shown to solve most of the outstanding problems, such as the interference and resolution limitations. Incoherent illumination in the NIR wavelength range (1.1‒1.7 μm) was applied to penetrate the Si substrate without interference from the substrate; however, the longest wavelength of the illumination limited the resolution. To address this issue, a combination of an SIL and the high refractive index of Si was used. Although this imaging technique proved to be a powerful means of overcoming the problem of diffraction-limited resolution, imperfections in the SIL shape and imperfect contacts between the SIL and sample caused aberrations in the image and a reduction in the numerical aperture (NA) of the system, respectively. Furthermore, the SIL could not be used to inspect flip-chip bonded devices in many cases because each new application required a new design with the optimum geometry and constituent material for each sample. In another previous work on improving the image contrast and SNR of the system, the authors demonstrated a time gating imaging technique using the second harmonic generation phenomena to reduce the strong reflection from the front of the substrate without requiring complex sample preparations, such as unpacking, polishing, and antireflection coating [10]. The experimental setup consisted of a coherent infrared pulsed laser (1.03 μm) and a correlator system that included a non-linear optical crystal, which enabled the detection of a laser beam that was reflected from only the layer of interest in the device. However, this approach had a low spatial resolution of 10 μm due to the absence of an objective lens. Finally, a confocal thermoreflectance imaging technique was implemented to achieve high-contrast depth-resolved thermal imaging [11]. The technique was based on a Nipkow disk confocal microscope, which provides an optical sectioning capability to suppress extraneous reflections from above or below the imaging plane. It was demonstrated that the system enabled improvement of the dynamic range and thermal sensitivity for thermal imaging of an encapsulated or semi-obstructed device. However, Nipkow disk confocal microscopes have drawbacks such as very low light transmission through the pinhole on the spinning disk.

In this paper, we propose a confocal TRM system that achieves high-sensitivity, high-resolution thermoreflectance measurements in the backside thermal imaging of various microelectronic devices. We describe the development of the confocal TRM system based on a laser scanning confocal microscope using galvanometer scanners and a Fourier-domain filtering method in detail. The main aspects of our backside thermal imaging system are a confocal imaging technique for isolating extraneous reflections and a Fourier-domain filtering method for performing quasi-point thermoreflectance measurements on the laser scans of the sample. To validate the improved measurement sensitivity and spatial resolution of the system, we compared the experimental results of the proposed laser scanning confocal TRM with those of a conventional widefield TRM in topside and backside thermal measurements.

## 2. Experimental Methodology

### 2.1. Experimental Setup

**Figure 1:**
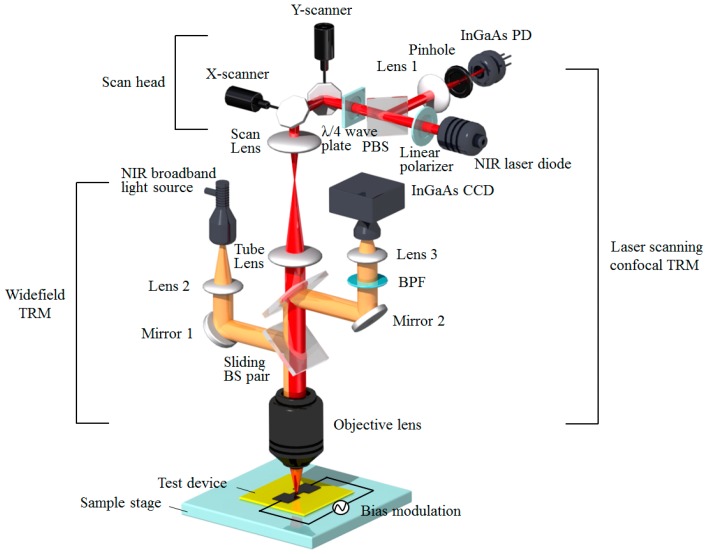
Schematic diagram of the NIR laser scanning confocal TRM system. The system also includes a widefield TRM system. Photodiode: PD, Polarizing beam splitter: PBS, Bandpass filter: BPF, Beam splitter: BS.

The developed experimental setup for the proposed confocal TRM system is presented in Figure 1. The system is based on a laser scanning confocal microscope, which is combined with a CCD-based widefield microscope, and provides a direct means of comparing the thermoreflectance measurements obtained using the two methods.

The laser scanning confocal microscope primarily consists of a scan head, two relay lenses (a scan lens and a tube lens), and an objective lens. The scan head consists of *x*- and *y*-axis galvanometric scan mirrors, a *λ*/4 wave plate, a polarizing beam splitter (PBS), and a linear polarizer. An NIR laser diode with an 1150 nm wavelength (QFBGLD-1150-300, Qphotonics, Ann Arbor, MI, USA) is used as a coherent illumination source for the quasi-point thermoreflectance measurements passing through the Si substrate of the device under test (DUT). Two galvanometric scan mirrors (VM500+, Cambridge technology, Bedford, MA, USA) are employed to accomplish both fast horizontal (*x*-axis) and slower vertical (*y*-axis) scans on the sample plane. The linear polarizer (LPNIRE050-B, Thorlabs, Newton, NJ, USA) and the *λ*/4 wave plate (AQWP05M-980, Thorlabs) are used to adjust the intensity and polarization of the transmitted laser beam, respectively. A combination of the *λ*/4 wave plate and PBS (CM05-PBS203, Thorlabs) is used to deflect the laser beam efficiently with minimal loss because the polarization of the laser is rotated by 90° with respect to its initial direction after passing through the *λ*/4 wave plate twice. An important issue when constructing a laser scanning microscope is that the laser beam that enters the back aperture of the objective lenses (50× & NA 0.42, 100× & NA 0.5, M Plan Apo NIR, Mitutoyo, Kanagawa, Japan) should be stationary during the *x*-y scanning process [12]. To ensure that this requirement is met, the scan lens (*f* = 40 mm, MPM-SL, Thorlabs) and tube lens (*f* = 200 mm, MPM-SL, Thorlabs) are separated by the sum of their focal lengths to act as an intermediate telecentric system. Then, the centers of the two scan mirrors and the back focal plane of the objective lens are approximately positioned at the telecentric planes formed by the two relay lenses (scan and tube lenses). The objective lens focuses the laser beam on the DUT and collects the reflected light from the DUT. The reflected laser beam is then transmitted back toward the PBS after passing through the *λ*/4 wave plate and is deflected into a detector as a result of the polarization change during the round-trip reflection. The detector consists of Lens 1, a pinhole (30 μm or 50 μm diameter), and an InGaAs photodiode (DET01CFC, Thorlabs), as shown in Figure 1. The pinhole diameter is set to the optimal value of ~0.5 Airy units depending on the objective lens in use [13]. To control the scanner and acquire images, a data acquisition board (NI PXIe-6361, National Instruments, Austin, TX, USA) is used to provide synchronized sinusoidal and sawtooth waveforms as the driving signals for the *x*- and *y*-axis scanning mirrors, respectively. Subsequently, the digitizer (14-bit, NI PXIe-5122, National Instruments) samples the signal level of the light detected by the confocal detector and converts it into a digital format. Before sampling, the detected light signal is enhanced using an amplifier (SR570, Stanford Research System, Sunnyvale, CA, USA). Of all the data that are acquired during the laser scan, only those obtained during a half period of an *x*-axis scan, namely, the forward-direction scan, are chosen and arranged two-dimensionally for image acquisition. However, all of the data acquired during bi-directional scans can be used to obtain higher image frame rates. This process is made possible by the precise synchronization between the two mirrors. The widefield microscope consists of an NIR broadband light source (MHAB-100W-IR, MORITEX, Saitama, Japan), a sliding beam splitter pair, a 10 nm bandpass filter with a center wavelength of 1150 nm (2-1155, Optometrics Corporation, Littleton, MA, USA), and an InGaAs CCD camera (12-bit, NIR-300FCL, ALLIED Vision Technologies, Exton, PA, USA). The widefield microscope is used to observe the sample position during the experimental preparation but can then be used to obtain the backside thermal image using the conventional widefield TRM method. The beam splitter pair can be moved back and forth depending on the selected microscope. A custom-written LabVIEW program was created to implement the laser beam scanning control and image-processing program that managed the reflection and thermoreflectance imaging modes in the two microscopes.

### 2.2. Sample Description

**Figure 2:**
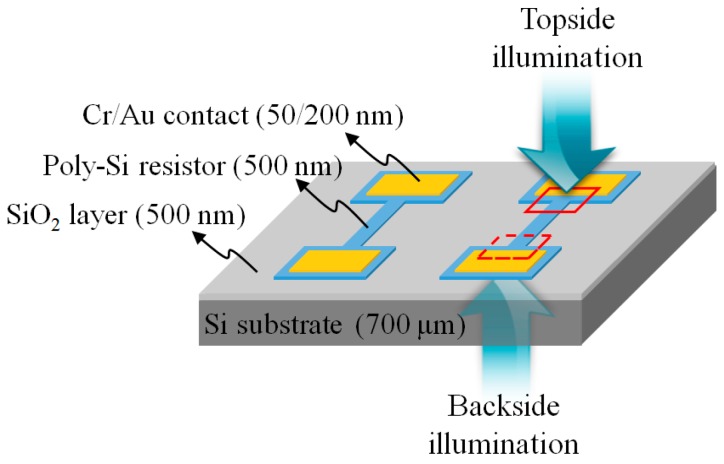
DUT design with a poly-Si micro resistor on a SiO_2_ layer/Si substrate and the illumination directions for topside and backside measurements.

Figure 2 illustrates the design of a micro heater that was used as the DUT and also shows the illumination directions for the topside and backside measurements. The micro heater was fabricated using a thin-film polycrystalline Si (poly-Si) resistor with Cr/Au ohmic contacts on a SiO_2_ layer/Si substrate. The poly-Si micro resistor was 20 μm wide by 200 μm long, the resistance was about 1 kΩ and the Si substrate was 700 μm thick. Using a wire bonding method, the Cr/Au ohmic contacts were interconnected with the pads on the custom-designed printed circuit board (PCB) after the DUT was put on the PCB. Note that it is convenient to supply a bias signal while the DUT is upside-down for backside measurements. When using the confocal TRM system, the temporally modulated bias signal to the micro resistor was provided by a radio-frequency power amplifier (WMA-300, Falco Systems, Katwijk aan Zee, The Netherlands) with a seed signal from the waveform generator (PXI-5422, National Instruments).

### 2.3. Thermoreflectance Imaging Technique

**Figure 3:**
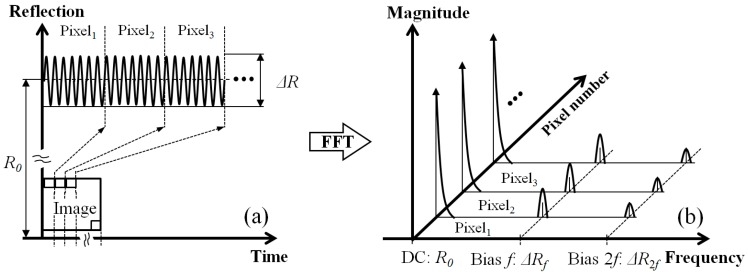
(**a**) Individual data set for the time-varying reflected laser beam as a single pixel and (**b**) reflectivity variations in the frequency domain after the FFT.

When a bias modulation signal at a frequency *f* was supplied to the DUT, temperature modulation was generated by the dissipated power *P* [14]. The scheme of thermoreflectance imaging in TRM is based on detecting the reflectivity variation Δ*R* with respect to the temperature modulation of the DUT. In our previous study, a Fourier-domain filtering method for CCD-based TRM was introduced as a thermoreflectance image acquisition strategy and offered high-sensitivity thermoreflectance imaging at a faster imaging speed than the conventional four-bucket method [15]. Here, the Fourier-domain filtering method was conceptually applied to laser scanning confocal TRM but was implemented in different ways as follows. In the first step, the time-varying reflected laser beams from the bias-modulated DUT are detected by the single photodiode along with the sampling rate during the laser scan. In the second step, the serial data acquired during the forward-direction scan are separated into individual data sets that are matched for a single pixel in the image and then each data set is converted into the frequency domain by performing a fast Fourier transform (FFT), as shown in Figure 3a,b. In the final step, the peak values of the bias modulation frequency component Δ*R_f_* and the dc component *R*_0_ are extracted from each data set in the frequency domain and the calculated relative reflectivity variations Δ*R_f_*/*R*_0_ for each data set are presented as pixels in the image Δ*R*/*R* (*x*, *y*).

### 2.4. System Configuration Characteristics

It is worth noting the system configuration for backside thermal imaging in confocal thermoreflectance measurements. In a previous study, a Nipkow disk confocal microscope was used to obtain high-contrast thermal images of encapsulated devices for comparison with widefield measurements [11]. However, even though the disk was antireflection-coated, a small proportion of the illumination was reflected by the surface of the disk. These reflections could become high-level background noise because the light transmitted through the pinhole arrays on the disk was very low. In another previous study, a laser scanning thermoreflectance imaging system using galvanometric mirrors was proposed for faster surface temperature measurements than those of the point classical thermoreflectance technique [14]. In contrast, in our system, a dual-axis galvanometer scanning head with a pinhole in the detector was adopted to facilitate the achievement of high confocality without the drawbacks of spinning disks in sub-surface thermal measurements through the substrate. In addition, we devised a Fourier-domain filtering method to match the scanning thermoreflectance measurements, which differs from the image-based, four-bucket method and lock-in amplifier detection described in References [11,14], respectively. In particular, this method eliminates the need for phase locking between the detection equipment and the bias modulation signal of the sample.

## 3. Results and Discussion

### 3.1. Image Acquisition

**Figure 4:**
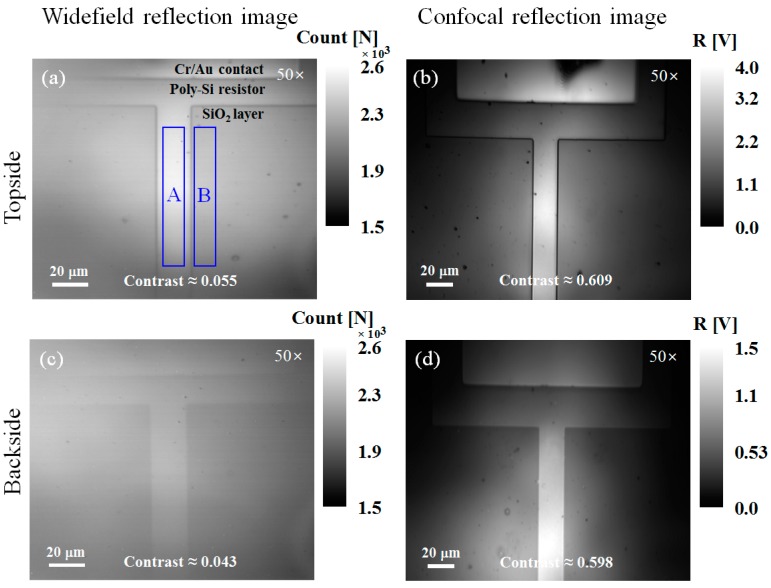
Topside and backside reflectance (*R*) images of the poly-Si micro resistor obtained by (**a**,**c**) the widefield microscope and (**b**,**d**) the confocal microscope with a 50× objective lens (NA 0.42).

To test the image acquisition performances of the widefield and confocal microscopes, the reflection images of the fabricated device were obtained by a 50× objective lens (NA 0.42). The 30-μm-diameter pinhole was employed in the confocal image acquisition using the 50× objective lens. Figure 4a,c and Figure 4b,d show the widefield and confocal reflection images obtained using topside and backside illumination, respectively. The image sizes were 175 × 140 μm^2^ with 320 × 256 pixels and 200 × 160 μm^2^ with 500 × 400 pixels for the widefield and confocal microscopes, respectively. The contrast in Figure 4 was defined as (*I_A_* − *I_B_*)/*I_B_*, where *I_A_* and *I_B_* are the average of the reflection signals in regions A and B, respectively. Because the NIR source light could partially pass through the layers configured in the sample, the extraneous reflections from the interface between the air and Si substrate occurred in both the topside and backside illumination cases. For the reflection images obtained by the widefield microscope that are shown in Figure 4a,c, the contrast in the backside image is reduced by ~20% compared to that in the topside image. This reduction occurred because the extraneous reflections in the backside illumination case were more strongly reflected by the interface. On the other hand, the contrast in the backside image obtained by the confocal microscope is not reduced compared to that in the topside image. In addition, the image contrasts in the confocal microscope are greater than that in the widefield microscope. This increased contrast results from the optical sectioning capability that eliminates the strong obstructive reflections from the front of the Si substrate. During backside image acquisition, wave interference between the light reflected from the front and bottom of the device substrate occurred. The interference between waves becomes stronger when the coherent length (*l_c_* ≈ *λ*^2^/*n*·Δ*λ*) of the illumination source is longer than the difference between the path lengths of the two reflection beams [16], which is equal to twice the thickness of the device substrate. In the confocal microscope, the coherent length (~3.7 mm) of the NIR laser diode—which had a wavelength of *λ* = 1150 nm and a line width of Δ*λ* = 0.1 nm—was much longer than twice the substrate thickness of 700 μm. Here, the Si substrate had a refractive index of *n* = 3.56 at 1150 nm. Thus, the use of coherent illumination (i.e., the NIR laser source) resulted in interference fringes in the backside imaging. However, the confocal microscope with the pinhole provided backside images without interference patterns due to its optical sectioning capability, as shown in Figure 4d. Therefore, we expect that the confocal microscope can improve the image contrast and reduce the interference in backside imaging through the substrate.

**Figure 5:**
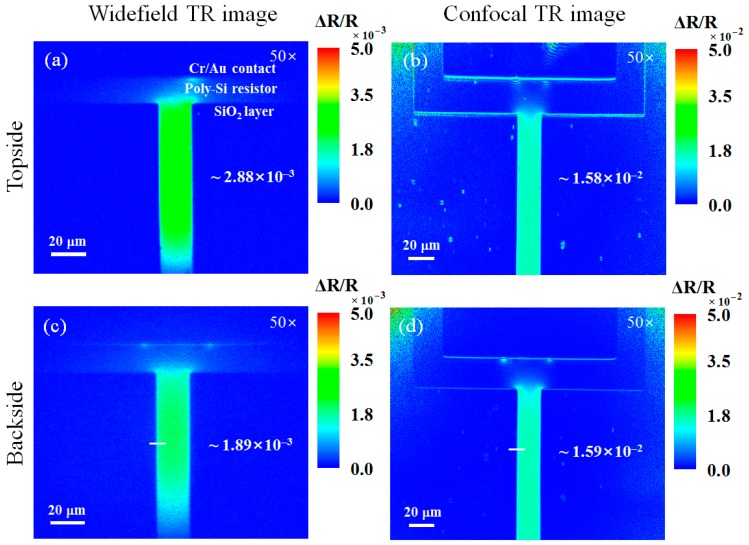
Topside and backside Δ*R/R* images of the poly-Si micro resistor that were obtained using (**a**,**c**) the widefield TRM and (**b**,**d**) the confocal TRM with a 50× objective lens (NA 0.42). The numbers in (**a**–**d**) indicate the Δ*R/R* values of the micro resistor. The solid lines in (**c**,**d**) indicate the locations of the profile shown in Figure 6.

Figure 5 shows topside and backside thermoreflectance images of the poly-Si micro resistor obtained by the widefield and confocal TRM systems using a 50× objective lens (NA 0.42). Some localized heat generation occurs at the interface between the Cr/Au contact pad and poly-Si micro resistor. In a previous study, we used a scanning electron microscope to confirm that the localized hot spots in the thermoreflectance images originate from physical defects, which cause local contact resistance variations and thereby generate localized Joule heating [17]. In the confocal TRM system, the micro resistor was supplied by a 100-kHz sine voltage varying from 0 V to 20.4 V. During the image acquisition, the sampling rate was 5 MS/s (mega samples per second) and the pixel dwell time and image resolution were ~100 μs and 500 × 400 pixels, respectively. Consequently, the acquisition time of the thermoreflectance image was 20 s. For the performance comparison, a widefield thermoreflectance image was obtained by the widefield TRM system combined with the confocal TRM system. The same bias conditions that generated the equivalent reflectivity variation Δ*R* were applied to the DUT, except for the slow bias modulation frequency of 4 Hz due to the slow frame rate of the CCD camera used in the widefield TRM system. To ensure that the comparison was performed using the same measurement time, reflection images were acquired for 20 s in the widefield TRM system and the acquired images were then averaged to obtain a single thermoreflectance image. One limitation of our confocal TRM system is that the acquisition time depends on the bias modulation frequency range. The higher the bias modulation frequency, the shorter the acquisition time. However, the bias modulation frequency cannot be increased infinitely because the magnitude of the thermal signal would gradually be reduced.

### 3.2. Confocal Thermoreflectance Measurement Characteristics: Resolution and Sensitivity

**Figure 6:**
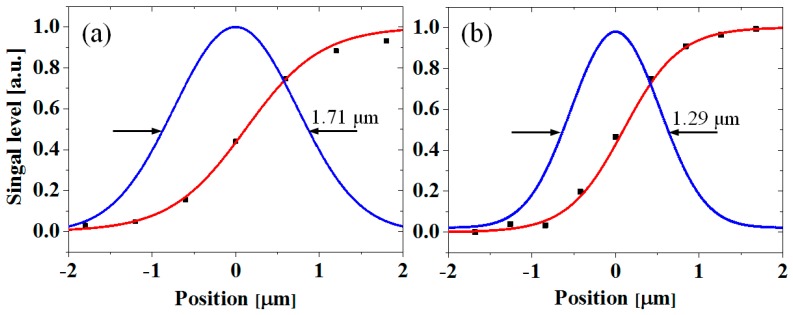
Spatial resolutions of backside thermoreflectance measurements using the (**a**) widefield and (**b**) confocal TRM systems with a 50× objective lens (NA 0.42). The symbols (solid squares) are the experimental data indicated by the solid lines in Figure 5c,d and were fitted to error functions (red lines). Gaussian PSFs (blue lines) were extracted based on the derivatives of the fitted curves. The position was measured from the edge of the poly-Si micro resistor.

First, we quantified the spatial resolutions of the widefield and confocal TRM systems in the backside thermoreflectance measurements by taking a line profile across the edge of the poly-Si micro resistor, as shown in Figure 5c,d. The sampled data for each case was fitted to an error function before a Gaussian point-spread function (PSF) was extracted based on the derivative of the fitted curve. The spatial resolution was defined as the full-width at half-maximum of the Gaussian PSF [18,19]. Figure 6a,b illustrates the spatial resolutions of 1.71 μm and 1.29 μm that were achieved for the widefield and confocal TRM systems, respectively, using a 50× objective lens (NA 0.42). This comparison illustrates the ~25% spatial resolution improvement provided by the developed confocal TRM system, as compared to the widefield TRM system.

Second, we confirmed a method of evaluating the sensitivity improvement of the backside thermoreflectance measurements using Δ*R*/*R* between the widefield and confocal thermoreflectance images. The sensitivity for imaging small temperature changes in TRM is directly related to the thermoreflectance coefficient *κ* and the bit depth in the image acquisition [1]. While it is conceptually straightforward to compare the sensitivities of widefield and confocal thermoreflectance measurements in terms of *κ*, such comparison is not simple in practice. Generally, the *κ* measurement is based on the iterative acquisition of reflection images as the temperature of the sample increases in accordance with the procedure in Reference [18]. However, when this procedure is followed for backside *κ* measurement, the ROI of the sample can become contaminated because the upside-down sample is attached to a thermoelectric cooler element. Thus, we chose to compare Δ*R*/*R* to determine the measurement sensitivity, which is proportional to *κ* when the same temperature variation Δ*T* in the sample occurs in two measurements obtained by different methods, as shown in Equation (1). To validate the sensitivity improvement evaluation using Δ*R*/*R*, the topside and backside Δ*R*/*R* images obtained by the widefield and confocal TRM systems were investigated. The images were acquired under the same bias conditions that generated the same Δ*T*. Topside and backside thermoreflectance images are shown in Figure 5a,b and Figure 5c,d, respectively. Δ*R*/*R* of the poly-Si micro resistor for each case in Figure 5 is indicated. Table 1 summarizes the illumination wavelength, *κ*, and Δ*T* for the topside thermoreflectance measurements of the poly-Si micro resistor. Each Δ*T* was calculated from the measured *κ*. The discrepancy between the topside and backside measurements shown in Figure 5a,c signifies that the widefield TRM system, unlike the confocal TRM system, was affected by the strong reflections from the front of the substrate. We checked that, although there are differences between Δ*R*/*R* of the poly-Si micro resistor in the widefield and confocal topside thermoreflectance measurements shown in Figure 5a,b, Δ*T* is are similar. Moreover, the ratio (5.5) between Δ*R*/*R* in Figure 5a,b and the ratio (5.8) between the *κ* values in Table 1 are almost in agreement. On this basis, we concluded that Δ*R*/*R* accurately represents the measurement sensitivity in place of *κ*.

In Table 1, *κ* for the confocal TRM is higher than that for the widefield TRM. This difference results from the optical sectioning capability of the confocal TRM, which enables the detection of small reflection changes only on the poly-Si micro resistor during measurement with topside illumination.

**Figure 7:**
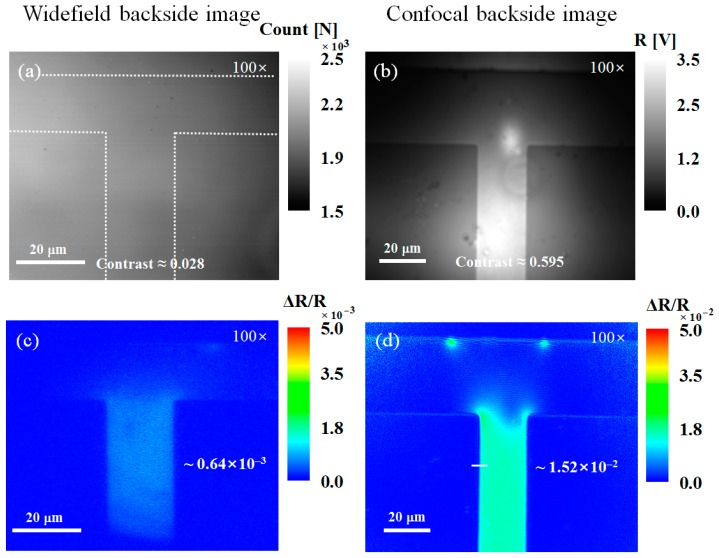
Backside reflection (top) and backside thermoreflectance (bottom) images of the poly-Si micro resistor obtained by (**a**,**c**) widefield and (**b**,**d**) confocal TRM systems with a 100× objective lens (NA 0.5), respectively. The dotted line in (**a**) represents the approximate region of the poly-Si resistor. The numbers in (**c**,**d**) indicate the Δ*R/R* values of the micro resistor. The solid line in (**d**) indicates the location of the profile shown in Figure 8.

**Figure 8:**
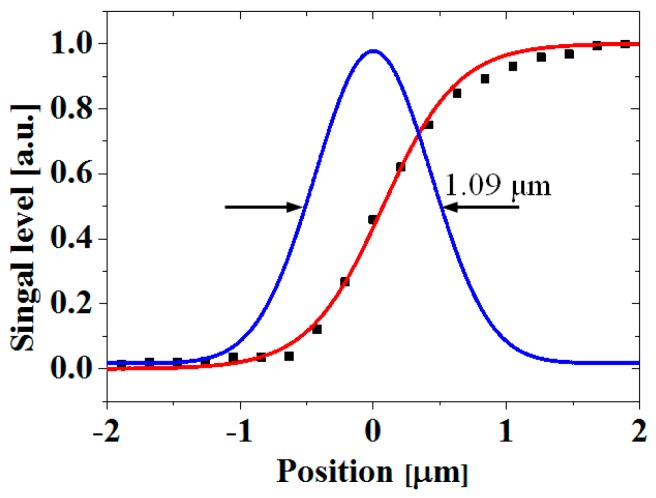
Spatial resolution of backside thermoreflectance measurements obtained using the confocal TRM system with a 100× objective lens (NA 0.5). The symbols (solid squares) are the experimental data indicated by the solid line in Figure 7d and were fitted to an error function (red line). A Gaussian PSF (blue line) was extracted from the derivative of the fitted curve. The positions were measured from the edge of the poly-Si micro resistor.

Finally, we demonstrated the sensitivity improvement for the backside thermoreflectance measurements using the developed confocal TRM system after increasing the imaging capability by using a high-magnification objective lens with a high NA. Figure 7 shows the backside reflection and backside thermoreflectance images obtained by the widefield and confocal TRM systems using a 100× objective lens (NA 0.5). As the incident angle of the objective lens increased, the reflection from the front of the substrate, which is a source of background noise, became stronger. Thus, the image contrast in the widefield backside image shown in Figure 7a is additionally decreased by ~35% from that of the backside measurement using the 50× objective lens. However, the confocal backside imaging with an optimized pinhole (50 μm diameter) was much more robust in the presence of strong reflections, as shown in Figure 7b,d. The spatial resolution of the confocal backside measurements in Figure 8 is also characterized by the confocal backside thermoreflectance image in Figure 7d. As a result, the confocal backside thermoreflectance measurements were found to exhibit a ~23-fold improvement in thermal sensitivity over those of the widefield systems, from the ratio between Δ*R*/*R* in Figure 7c,d and have a spatial resolution of ~1.09 μm, as shown in Figure 8.

## 4. Conclusions

This paper described the development of a confocal TRM system based on a laser scanning microscope and a Fourier-domain filtering method to improve the backside thermal imaging of microelectronic devices. In our system, confocal microscopy based on a fast galvanometer laser scanner was adapted to remove extraneous reflections from the Si substrate and Fourier-domain filtering was applied to provide a simple thermoreflectance measurement implementation. We then demonstrated that the confocal TRM system prevents out-of-focus reflections from degrading the thermal measurements and provides high-sensitivity, high-resolution backside thermoreflectance imaging. When compared to conventional CCD-based widefield TRM system, the laser scanning confocal TRM system, which takes advantage of both confocal detection and Fourier-domain processing, improves the backside thermal measurements in dissipative microelectronic devices. Therefore, we believe that the developed confocal TRM system is particularly valuable for investigating the thermal characteristics of microelectronic devices.

## Figures and Tables

**Table 1:** Summary of the illumination wavelength, *κ*, and Δ*T* for two methods of obtaining topside thermoreflectance images of the poly-Si micro resistor.

Method of Topside Thermoreflectance Measurement	Illumination Wavelength (nm)	*κ* (K^−1^)	Δ*T* (K)
Widefield TRM using four-bucket method	1150 ± 10	−1.79 × 10^−4^	16.1
Confocal TRM using Fourier-domain filtering method	1150	−1.04 × 10^−3^	15.2

## References

[1] Farzaneh M., Maize K., Lüerßen D., Summers J.A., Mayer P.M., Raad P.E., Pipe K.P., Shakouri A., Ram R.J., Hudgings J.A. (2009). CCD-based thermoreflectance microscopy: Principles and applications. J. Phys. D Appl. Phys..

[2] Mayer P.M., Lüerßen D., Ram R.J., Hudgings J.A. (2007). Theoretical and experimental investigation of the thermal resolution and dynamic range of CCD-based thermoreflectance imaging. J. Opt. Soc. Am. A.

[3] Kim D.U., Park K.S., Jeong C.B., Kim G.H., Chang K.S. (2016). Quantitative temperature measurement of multi-layered semiconductor devices using spectroscopic thermoreflectance microscopy. Opt. Express.

[4] Burzo M.G., Komarov P.L., Raad P.E. (2005). Noncontact transient temperature mapping of active electronic devices using the thermoreflectance method. IEEE Trans. Compon. Pack. Tech..

[5] Grauby S., Dilhaire S., Jorez S., Claeys W. (2003). Imaging setup for temperature, topography, and surface displacement measurements of microelectronic devices. Rev. Sci. Instrum..

[6] Singh R., Nurnus J., Bian Z., Christofferson J., Shakouri A. (2009). Temperature profile inside microscale thermoelectric module acquired using near-infrared thermoreflectance. IEEE Trans. Compon. Pack. Tech..

[7] Christofferson J., Shakouri A. (2004). Thermal measurements of active semiconductor micro-structures acquired through the substrate using near IR thermoreflectance. Microelectron. J..

[8] Tessier G., Bardoux M., Boué C., Filloy C., Fournier D. (2007). Back side thermal imaging of integrated circuits at high spatial resolution. Appl. Phys. Lett..

[9] Tessier G., Bardoux M., Filloy C., Boué C., Fournier D. (2007). High resolution thermal imaging inside integrated circuits. Sens. Rev..

[10] Rampnoux J.M., Michel H., Salhi M.A., Grauby S., Claeys W., Dilhaire S. (2006). Time gating imaging through thick silicon substrate: A new step towards backside characterization. Microelectron. Reliab..

[11] Summers J.A., Yang T., Tuominen M.T., Hudgings J.A. (2010). High contrast, depth-resolved thermoreflectance imaging using a Nipkow disk confocal microscope. Rev. Sci. Instrum..

[12] Pawley J.B. (2006). Handbook of Biological Confocal Microscopy.

[13] Kubitscheck U. (2017). Fluorescence Microscopy: From Principles to Biological Applications.

[14] Grauby S., Salhi A., Rampnoux J.-M., Michel H., Claeys W., Dilhaire S. (2007). Laser scanning thermoreflectance imaging system using galvanometric mirrors for temperature measurements of microelectronic devices. Rev. Sci. Instrum..

[15] Choi W.J., Ryu S.Y., Kim J.K., Kim D.U., Kim G.H., Chang K.S. (2013). High-speed thermoreflectance microscopy using charge-coupled device-based Fourier-domain filtering. Opt. Lett..

[16] Pedrotti F.L., Pedrotti L.S. (1993). Introduction to Optics.

[17] Ryu S.Y., Kim D.U., Kim J.K., Choi H.Y., Kim G.H., Chang K.S. (2015). Surface-temperature measurement and submicron defect isolation for microelectronic devices using thermoreflectance microscopy. Int. J. Thermophys..

[18] Tessier G., Polignano M.L., Pavageau S., Filloy C., Fournier D., Cerutti F., Mica I. (2006). Thermoreflectance temperature imaging of integrated circuits: Calibration technique and quantitative comparison with integrated sensors and simulations. J. Phys. D Appl. Phys..

[19] Ramsay E., Pleynet N., Xiao D., Warburton R.J., Reid D.T. (2005). Two-photon optical-beam-induced current solid-immersion imaging of a silicon flip chip with a resolution of 325 nm. Opt. Lett..

